# Hydrogen Peroxide Promotes Injury-Induced Peripheral Sensory Axon Regeneration in the Zebrafish Skin

**DOI:** 10.1371/journal.pbio.1000621

**Published:** 2011-05-24

**Authors:** Sandra Rieger, Alvaro Sagasti

**Affiliations:** Department of Molecular, Cell and Developmental Biology, University of California Los Angeles, Los Angeles, California, United States of America; University of Cambridge, United Kingdom

## Abstract

Functional recovery from cutaneous injury requires not only the healing and regeneration of skin cells but also reinnervation of the skin by somatosensory peripheral axon endings. To investigate how sensory axon regeneration and wound healing are coordinated, we amputated the caudal fins of zebrafish larvae and imaged somatosensory axon behavior. Fin amputation strongly promoted the regeneration of nearby sensory axons, an effect that could be mimicked by ablating a few keratinocytes anywhere in the body. Since injury produces the reactive oxygen species hydrogen peroxide (H_2_O_2_) near wounds, we tested whether H_2_O_2_ influences cutaneous axon regeneration. Exposure of zebrafish larvae to sublethal levels of exogenous H_2_O_2_ promoted growth of severed axons in the absence of keratinocyte injury, and inhibiting H_2_O_2_ production blocked the axon growth-promoting effects of fin amputation and keratinocyte ablation. Thus, H_2_O_2_ signaling helps coordinate wound healing with peripheral sensory axon reinnervation of the skin.

## Abstract 2

Production of H_2_O_2_ by injured zebrafish skin cells promotes the regeneration of nearby somatosensory axon terminals, thus coordinating wound healing of the skin with sensory reinnervation.

## Author Summary

Touch-sensing neurons project axonal processes that branch extensively within the outer layers of skin to detect touch stimuli. Recovering from skin injuries thus requires not only repair of damaged skin tissue but also regeneration of the sensory axons innervating it. To study whether skin wound healing is coordinated with sensory innervation, we compared the regeneration of severed sensory axons innervating larval zebrafish tail fins with and without concomitant injury to surrounding skin cells. Severed axons regenerated more robustly when nearby skin cells were also damaged, suggesting that wounded skin releases a short-range factor that promotes axon growth. The reactive oxygen species hydrogen peroxide (H_2_O_2_) is known to be produced by injured cells, making it a candidate for mediating this signal. We found that adding exogenous H_2_O_2_ improved the regeneration of severed axons. Conversely, blocking H_2_O_2_ production prevented the axon growth-promoting effect of skin injury. Thus, H_2_O_2_ promotes axon growth after skin damage, helping to ensure that healing skin is properly innervated.

## Introduction

Successful wound repair and regeneration requires coordination between the various cell types that make up the injured tissue. For example, following injuries that damage both epidermis and sensory endings, wounded epidermis promotes the regeneration of nerve fibers [1],[2]. Conversely, complete epidermal wound healing requires the presence of sensory axons [1],[3]. In amphibians, innervation of the wound epidermis by nerve fibers is also essential for limb regeneration and correlates with the establishment of signaling centers [4]–[6]. These observations imply that coordination between wound epidermis and sensory axons during healing and regeneration is regulated by molecular interactions between these cell types.

In mammals, peripheral axon regeneration is generally more robust than axon regeneration in the central nervous system. Nonetheless, reinnervation in the periphery can be slow or incomplete, depending on the extent of axonal injury and on interactions with surrounding cells [7],[8]. Because nerve injury is often associated with damage of not only the nerve but also neighboring tissues, it has been difficult to separate autonomous and non-autonomous factors influencing axon regeneration in vivo. Recent studies in *C. elegans* and zebrafish have utilized laser axotomy to precisely damage single axons in the peripheral nervous system, making it possible to assess the influence of non-neuronal tissues on axonal regeneration [9],[10].

Tissue damage triggers a complex cascade of signals that activate inflammatory responses and promote tissue repair [11]. In fruit flies and zebrafish, the recruitment of immune cells to wounds is mediated by the small reactive oxygen species (ROS) hydrogen peroxide (H_2_O_2_), which emanates from the injury [12],[13]. The role of H_2_O_2_ in oxidative stress has been well studied, as high levels can have deleterious effects on the maintenance of cell homeostasis [14]. In the nervous system, H_2_O_2_ can induce neurodegeneration through activation of pro-apoptotic pathways [15]–[17]. More recently it has come to be appreciated that H_2_O_2_ can act as a signaling molecule with specific developmental and physiological functions. H_2_O_2_ is thought to signal by oxidizing cysteine residues on target proteins, most notably phosphatases [18],[19].

The larval zebrafish tail fin provides an accessible setting for investigating how peripheral axon regeneration is coordinated with the healing of injured tissue and for testing whether H_2_O_2_ plays a role in these interactions. During larval stages, zebrafish fins consist of a folded two-layered epithelium, surrounding muscle cells (Figure S1A). Zebrafish tail fins regenerate after amputation, both during larval development [20],[21] and in adults [22], but sensory reinnervation of regenerated fins has not been explicitly assessed.

**Figure 1:**
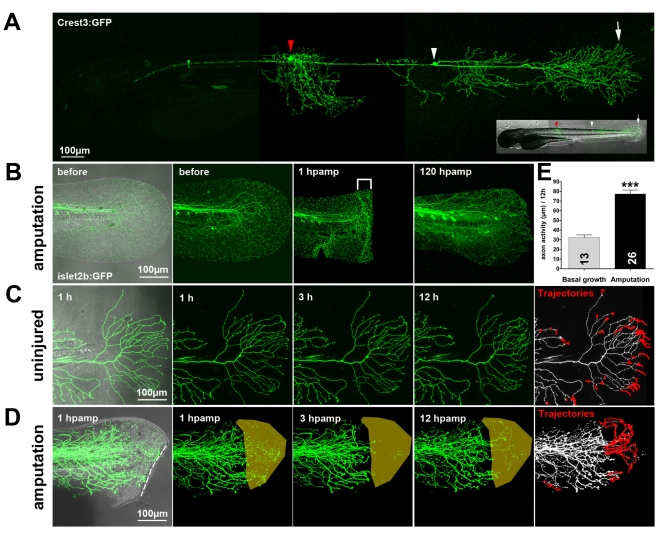
Amputation promotes peripheral sensory axon regeneration in the fin epidermis of 78 hpf zebrafish larvae. (A) A transient transgenic larva expressing GFP in two RB neurons: one innervating the trunk (red arrowhead) and one innervating the tail fin (white arrowhead). Arrow indicates the RB peripheral arbor in the tail fin. (B) Fin amputation in a 78 hpf islet2b:GFP transgenic larva caused severed axon branches to degenerate at the wound margin (brackets, 1 h post-amputation [hpamp]). RB axon arbors completely reinnervated the regenerated fin by 120 hpamp. (C, D) Time-lapse sequences from 78–90 hpf. The rightmost panel shows axon tip trajectories (red) over the course of the time-lapse (see also Video S1 for a tracing example). (C) The branches of a single GFP-labeled peripheral axon in an uninjured fin underwent minimal growth and retraction. Some of this apparent activity was due to movement of the tissue during time-lapse (see Video S1). (D) Fin amputation (dotted line) increased the growth of the severed arbor (see also Figure 3D, E) and promoted reinnervation of denervated territory (shaded area) (Video S3). (E) Quantification of axon activity in uninjured fins (*n* = 13) and after fin amputation (*n* = 26) (two-tailed, unpaired Student's *t*-test, *** *p*<0.001). Error bars represent the standard error of the mean. hpamp, hours post amputation.

Somatosensation at larval stages in zebrafish is accomplished by two populations of neurons: trigeminal neurons, which are located in ganglia outside the hindbrain and innervate the skin of the head, and Rohon-Beard (RB) neurons, which are located in the dorsal spinal cord and innervate the skin of the trunk and tail (Figure 1A). The peripheral axons of somatosensory neurons arborize between the two epithelial layers that make up the larval skin, the outer periderm and inner basal cell layers [23]. Precisely severing a trigeminal peripheral axon after arborization is complete (∼36 h post-fertilization, hpf) promotes some regenerative growth, but regenerating axons avoid their former territories and undamaged neighboring axons never sprout into these newly denervated areas [24].

We have investigated the relationship between tissue damage and peripheral axon regeneration, using injury to the larval zebrafish tail fin as an experimental paradigm. Amputating the fin promoted peripheral sensory axon growth, allowing the robust reinnervation of the newly regenerated fin. This axon regeneration-promoting effect could also be elicited by ablating a few keratinocytes anywhere in the body. H_2_O_2_ exposure mimicked the axon growth-promoting effect of keratinocyte damage, and morpholino-mediated knockdown of the H_2_O_2_-generating enzyme Duox1 inhibited axon growth-promotion by fin amputation. Thus, H_2_O_2_ produced by damaged keratinocytes promotes the reinnervation of healing skin by sensory axons.

## Results/Discussion

**Figure 2:**
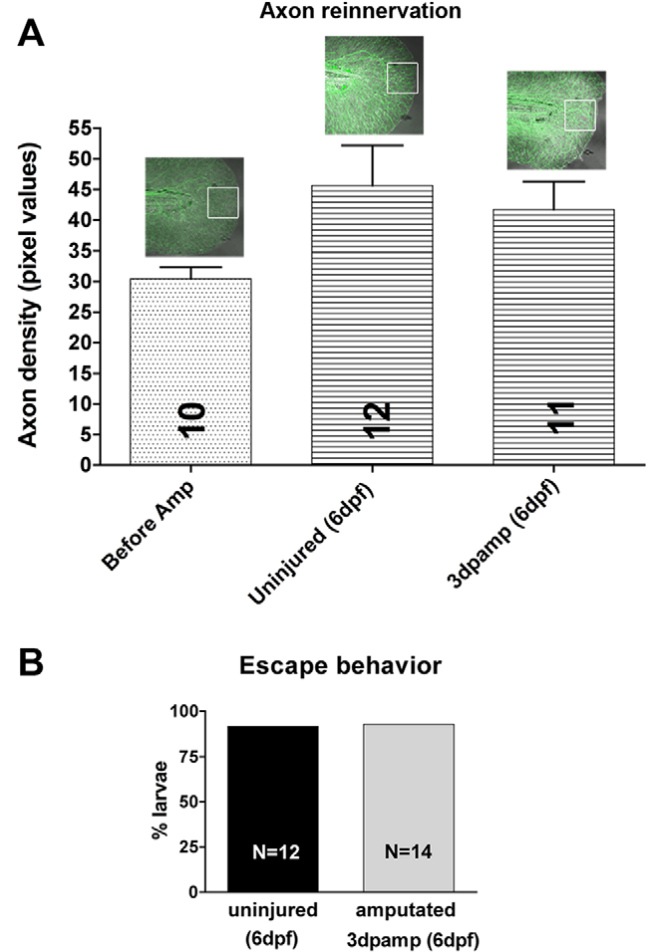
Sensory axons innervate regenerated fins. (A) Quantification of the density of axons within regenerated fins as measured by pixel density in a 50 µm^2^ area (white box) at the distal fin tip. No significant difference was found between groups (one-way ANOVA and Bonferroni's post-test for comparison of all groups was applied; *p* = *ns*>0.05). (B) Reinnervating sensory axons are functional. Uninjured larvae at 6 dpf were compared to amputated larvae at 3 dpa (6 dpf) in their ability to escape in response to touch stimuli at the caudal fin tip (11/12 uninjured fish escaped, 13/14 injured/regenerated fish escaped).

The caudal fins of larval zebrafish regenerate completely within a few days after amputation [20], implying that RB peripheral axons must also regenerate to provide sensory function to the new fin. To directly assess whether RB axons in the tail can regenerate, we imaged GFP-labeled RB arbors in the islet2b:GFP transgenic line [25] after caudal fin amputation at 3 d post-fertilization (dpf). Amputation caused immediate degeneration of axon branches near the wound (Figure 1B, brackets), creating a denervated zone that regenerating axons would need to traverse to fully innervate the regenerating fin. Despite this potential barrier, the fin was always reinnervated by RB arbors at 120 h post-amputation (hpamp) (Figure 1B). Three days after fin amputation, there was no detectable difference in the total amount of sensory axons in regenerated fin tips and fin tips of age-matched animals (6 dpf) that were never injured, indicating that reinnervation of regenerated fins was complete (Figure 2A). Sensory reinnervation of regenerated fins was functional, since 6 dpf fish with regenerated fins responded to touch at the tip of the tail as often as uninjured control fish (Figure 2B).

**Figure 3:**
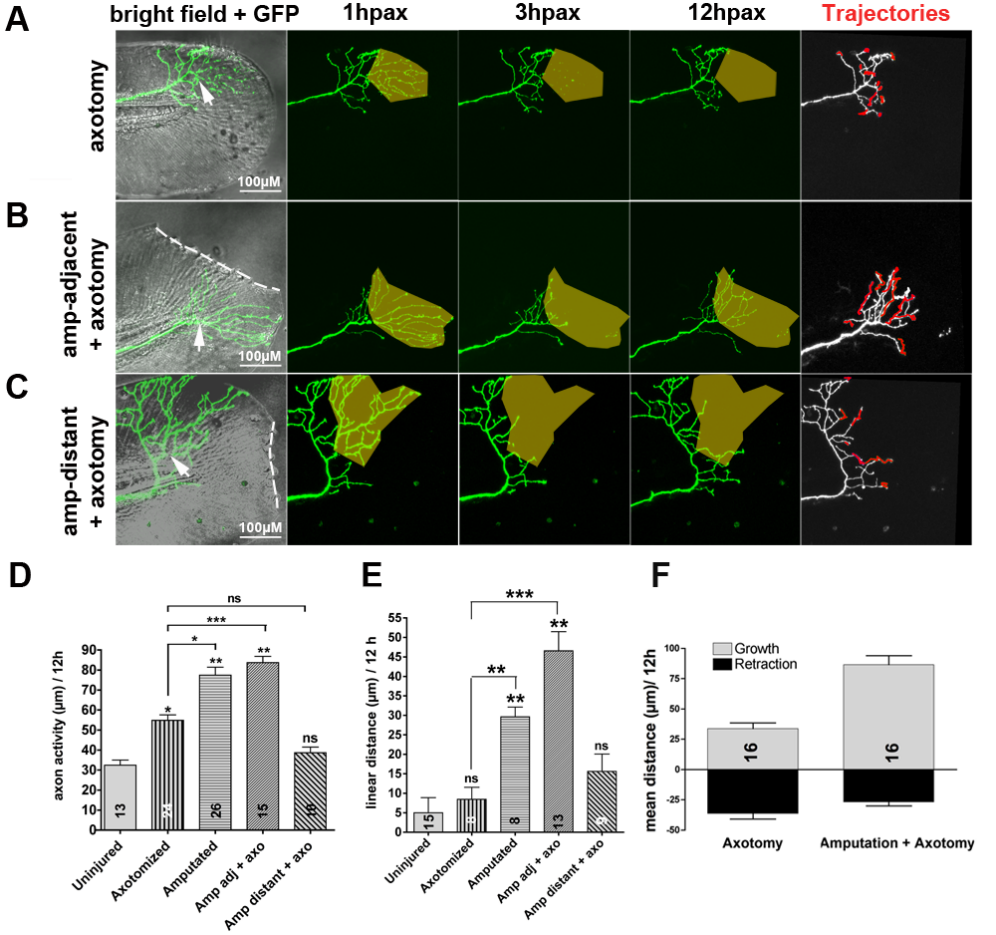
Signals from injured fins promoting axon regeneration function at short range. (A–C) Time-lapse sequences from 78–90 hpf. The rightmost panel shows axon tip trajectories (red) over the course of the time-lapse. (A) Axotomy (arrow) without amputation (see also Video S2). Axotomy increased axon activity, but axons were unable to reinnervate denervated territory (shaded area) (Figure S2). (B) The ability of an axotomized arbor (arrow) to reinnervate denervated territory (shaded area) was improved by fin amputation (dotted line) (see also Figure S2 and Video S4). (C) Axotomized arbors distant from the amputation plane did not regenerate (see also Video S5). (D, E) Comparison of axon activity (growth and retraction) (D) and linear growth distance (E) in uninjured arbors, after axotomy alone, after amputation alone, or after axotomy and amputation, both in close proximity (adj) or at a distance of greater than 50 µm. (F) Comparison of growth and retraction between axotomized axons, and axotomized axons in larvae whose fins were also amputated. Amputation in addition to axotomy promoted a shift from balanced growth and retraction toward growth. Sample size for each group is indicated by the number in the bar. Error bars represent the standard error of the mean. For statistical analyses, we performed one-way ANOVA and either Dunnett's post-test to compare individual groups to uninjured controls (asterisks above bars indicate significance compared to control) or Bonferroni's post-test to compare individual groups with each other (as indicated by brackets, *p* = *ns*>0.05, * *p*<0.05, ** *p*<0.01, *** *p*<0.001). hpax, hours post axotomy; amp, amputation.

The observation that RB axons robustly reinnervate larval fins within a few days after amputation, despite the fact that trigeminal axon regeneration is limited after precise axotomy [24], could be explained in either of two ways: (1) fin injury and healing promote peripheral axon growth or (2) RB neurons innervating the tail possess greater structural plasticity than trigeminal neurons. To assess the intrinsic plasticity of RB axon arbors, we monitored axon behavior after precise laser axotomy with time-lapse imaging for 12 h (see Figure S1B for experimental procedures) [10] and traced the position of individual axon tips every 30 min. Axotomy of RB neurons induced a 2-fold increase in axon activity (axon tip displacement, including both growth and retraction) compared to uninjured axons (54.92±2.72 µm, *n* = 24 versus 32.47±2.53 µm, *n* = 13 axon tip displacement, * *p*<0.05; compare Figures 1C and 3A; quantification in Figure 3D, Videos S2 and S1, respectively), but, like trigeminal axons, regenerating RB axons avoided denervated territory (Figure S2) [24]. Notably, axon growth was balanced by retraction, so that total arbor size did not substantially increase (Figure 3F; see Video S2). Like trigeminal axons [24], the ability of RB axons to reinnervate former territory in the fin was improved by inhibiting Rho kinase (unpublished data). Thus, the ability of RB axons to regenerate after fin amputation is likely not due to intrinsic regenerative capacity but is probably a specific response to tissue damage.

To further investigate the influence of tissue injury on RB axon regeneration, we compared the behavior of uninjured axon arbors (Figure 1C), precisely axotomized arbors (Figure 3A), and injured arbors in amputated fins (Figure 1D). Fin amputation (Video S3) increased total axon activity (growth and retraction) more than axotomy alone (77.40±4.03 µm, *n* = 26, *** *p*<0.001). Measuring the linear distance between an axon tip's position just after amputation and its position 12 h later revealed that fin amputation promoted productive axon growth, since axon tips traveled farther after amputation than after precise axotomy (29.62±2.50 µm, *n* = 8, versus 8.42±3.09 µm, *n* = 8, ** *p*<0.01; Figure 3E). Combining fin amputation with subsequent laser axotomy of a nearby RB axon branch increased the axon activity (83.74±3.09 µm, *n* = 26, ** *p*<0.01) and total growth (46.54±4.92 µm, *n* = 13, *** *p*<0.001) even further (Figure 3B,D,E, Video S4), but the amount of retraction was not dramatically altered (Figure 3F). Amputating fins significantly improved the ability of regenerating axons to innervate denervated areas (14.11±7.02 µm, *n* = 8 versus 60.24±13.06 µm, *n* = 10, * *p*<0.05; Figure S2), which is likely important for allowing regenerating arbors to traverse the denervated zone that forms just proximal to the wound after amputation (Figure 1B, brackets). Thus, fin injury increases sensory axon activity, promotes growth (but not retraction), and allows axons to overcome their avoidance of denervated territories.

To determine the effective range of axon growth-promoting signals from injured tissue, we axotomized axons distant (>50 µm) from the amputation site (Figure 3C, Video S5). These axons did not grow significantly better than precisely severed axons in uninjured tissue, since neither axon activity nor linear growth distances were increased by distant amputation (axon activity: 38.67±2.85 µm, *n* = 10, *p* = *ns*>0.05; linear distance = 15.63±4.42 µm, *n* = 9; *p* = *ns*>0.05; Figure 3D,E). Thus, growth-promoting signals emanating from injured tissue likely function at short range. To define the time window during which axons can respond to regeneration-promoting signals, we axotomized RB arbors at different time points after amputation. Axon activity was most enhanced when arbors were axotomized at 3 h post-amputation (114.6±7.04 µm, *n* = 10, ** *p*<0.01), but axotomy at 6 h post-amputation did not increase axon activity (61.20±6.45 µm, *n* = 10, *p* = *ns*>0.05; Figure S3A), as compared to axotomy alone. This observation suggests that axon growth-promoting signals are transiently emitted from the wound, rather than continuously from regenerating fin tissue. To assess whether the size of the severed arbor fragment influenced the amount of axon activity induced by amputation, we traced degenerated fragments in three dimensions to measure their total length and plotted length as a function of axon activity. Size of the axotomized arbor did not correlate with axon activity (Figure S3B).

**Figure 4:**
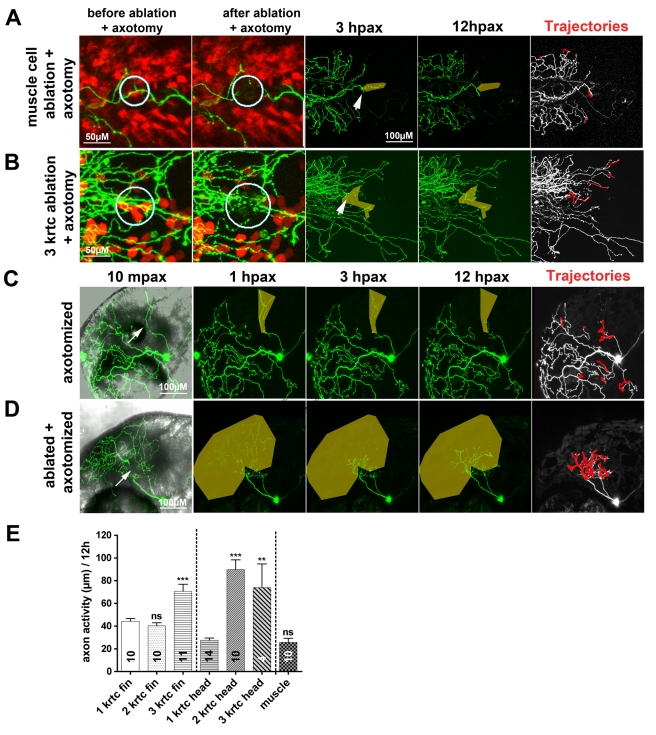
Keratinocyte damage promotes axon regeneration. Time-lapse sequences from 78–90 hpf. The rightmost panel shows axon tip trajectories (red) over the course of the time-lapse. (A) Ablating muscle cells (circles) in the fin of a transgenic reporter larva was accompanied by only limited regeneration of an axotomized arbor (arrow). (B) Ablating ≥3 keratinocytes (red) (circles) in the fin promoted axon regeneration after axotomy (arrow) and improved reinnervation of denervated territory (shaded area). (C) Axotomy of a trigeminal axon branch in the head (arrow) induced limited growth of the severed axon, but the denervated territory was avoided (shaded area). (D) Ablation of ≥3 keratinocytes and axotomy of a trigeminal axon (arrow) promoted robust growth of the severed axon and reinnervation of the denervated territory (shaded area). (E) Quantification of axon activity after keratinocyte and muscle cell ablations. Sample size for each group is indicated by the number in the bar. Error bars represent the standard error of the mean. For statistical analyses, we performed one-way ANOVA and Dunnett's post-test to compare individual groups to control groups (ablation of 1 keratinocyte in the fin or the head) (asterisks above bars indicate significance compared to control) (*p* = *ns*>0.05, ** *p*<0.01, *** *p*<0.001). krtc, keratinocyte.

To identify the origin of axon growth-promoting signals, we ablated individual muscle cells or keratinocytes in the fin of larvae expressing cell type-specific reporter transgenes that highlight each tissue [26],[27]. Ablating muscle cells did not promote axon growth (25.72±3.65 µm, *n* = 10; Figure 4A,E), but ablating ≥3 keratinocytes prior to axotomy provoked robust axon regeneration in both the fin (70.75±6.14 µm, *n* = 11, Figure 4B,E) and head (73.81±20.95 µm, *n* = 4; Figure 4C,D,E). However, ablating a single keratinocyte in either the fin (44.34±2.35 µm, *n* = 10, *** *p*<0.001) or the head (27.62±1.94 µm, *n* = 14, ** *p*<0.01) did not promote axon regeneration. This result suggests that a threshold of injury-induced signals is required to promote growth and reinnervation by RB and trigeminal axons.

**Figure 5:**
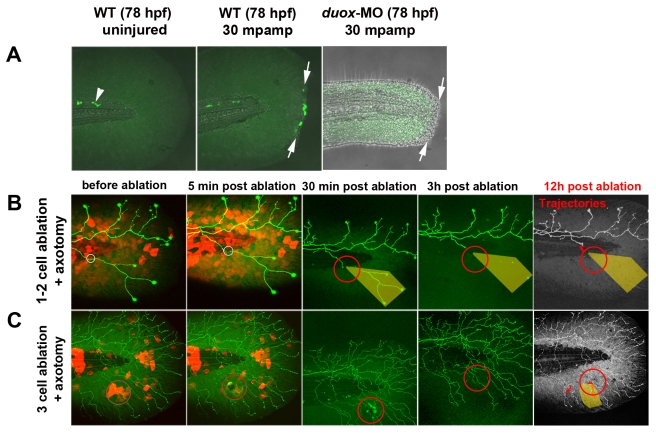
H_2_O_2_ detection following fin amputation at 78 hpf. (A) In uninjured fins, H_2_O_2_ was always detectable with pentafluorobenzenesulfonyl fluorescein (see Methods) in a few cells near the notochord (arrowhead) but was not detectable in fin tissue. Fin amputation (arrows) produced high levels of H_2_O_2_ in wound marginal cells at 30 min post-amputation. In contrast, H_2_O_2_ levels were either undetectable or very low in *duox1* morphants at 30 mpamp. (B, C) Ablating multiple keratinocytes produced hydrogen peroxide (H_2_O_2_). Time-lapse sequences from 78–90 hpf. The rightmost panel shows axon tip trajectories (red) over the course of the time-lapse and denervated territory (shaded areas). (B) Ablation of one or two keratinocytes (circles) did not produce sufficient H_2_O_2_ to be detected by the H_2_O_2_ sensor pentafluorobenzenesulfonyl fluorescein and did not promote axon regeneration of a nearby severed axon. (C) Ablating more (≥3) keratinocytes (circles) induced H_2_O_2_ production around the wound margin, as detected with the H_2_O_2_ sensor, and improved regeneration of a nearby axotomized arbor, which grew into denervated territory (shaded area, trajectories). mpamp, minutes post amputation.

The recently reported observation that zebrafish larval fin amputation produces high levels of hydrogen peroxide (H_2_O_2_) at the wound margin [13] prompted us to investigate whether H_2_O_2_ contributes to the promotion of axon regeneration by keratinocyte injury. By monitoring H_2_O_2_ with a chemical sensor (pentafluorobenzenesulfonyl fluorescein), we first verified that, like fin amputation (Figure 5A), laser ablating several keratinocytes produced detectable levels of H_2_O_2_ around the wound (Figure 5B). Ablation of 1–2 keratinocytes did not produce levels of H_2_O_2_ sufficient to detect with the chemical sensor, but ablating ≥3 keratinocytes generated detectable levels of H_2_O_2_ at the wound margin (Figure 5C), indicating that the severity of the injury correlates with the amount of H_2_O_2_ produced.

**Figure 6:**
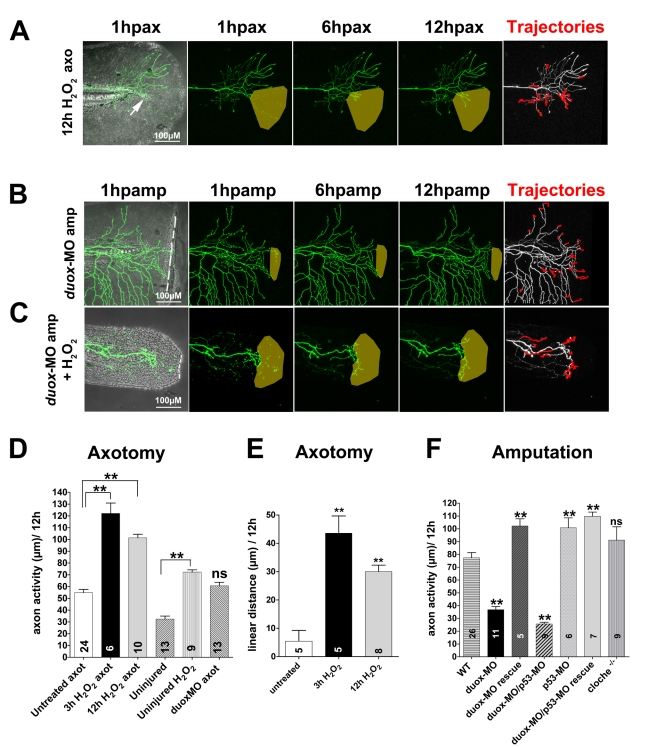
H_2_O_2_ promotes peripheral sensory axon growth in the skin. Time-lapse sequences from 78–90 hpf. The rightmost panel shows axon tip trajectories (red) over the course of the time-lapse; denervated territories are indicated by shaded areas. (A) Adding 3 mM H_2_O_2_ to the larval media for 12 h enhanced axon growth and promoted reinnervation of denervated territory after axotomy (arrow) in a non-amputated fin (see also Video S6). (B) Fin amputation did not promote axon growth in a *duox1*-morphant larva. The axon trajectories reflect mostly tissue movement during the time-lapse (see Video S7). (C) Adding 1.5 mM H_2_O_2_ rescued axon growth and improved reinnervation of denervated territory in amputated *duox1*-morphants (see also Video S8). (D, E) Quantification of axon activity (D) and linear axon growth (E) after axotomy in larvae treated with H_2_O_2_. (F) Quantification of axon activity after fin amputation. Sample size for each group is indicated by the number in the bar. Error bars represent the standard error of the mean. For statistical analyses, we performed one-way ANOVA and either Dunnett's post-test to compare individual groups to controls (asterisks above bar indicate significance compared to control, the first column in each graph) or Bonferroni's post-test to compare individual groups with each other (as indicated by brackets, *p* = *ns*>0.05, ** *p*<0.01). axo, axotomy; amp, amputation.

To test whether H_2_O_2_ can promote axon regeneration, we added 3 mM H_2_O_2_ (0.01%) to the larval media (the highest concentration of H_2_O_2_ at which most embryos survived and developed normally, see Figure S4 for survival rates) (Figure 6A, Video S6). The addition of H_2_O_2_ to uninjured larvae significantly promoted some axon activity (untreated, uninjured: 32.47±2.53 µm versus H_2_O_2_ uninjured: 72.30±1.94 µm, *** *p*<0.001; Figure 6D). Adding H_2_O_2_ for 3 or 12 h to larvae in which RB axon arbors had been axotomized increased axon activity variably but significantly, compared to axotomy in untreated animals (3 h H_2_O_2_: 122.1±8.81 µm, *n* = 6; 12 h H_2_O_2_: 101.4±3.09 µm, *n* = 10, versus untreated 54.92±2.72 µm, *n* = 24, ** *p*<0.01 each; Figure 6D). The linear growth distances of axotomized arbors were also increased by H_2_O_2_ (3 h: 43.58±6.06 µm, *n* = 5; 12 h: 30.04±2.25 µm, *n* = 8, versus untreated: 5.46±3.78 µm, *n* = 5, ** *p*<0.01 each; Figure 6E). Thus, H_2_O_2_ is sufficient to promote axon regeneration and does not need to be present in a gradient for this effect, as has been proposed for its role in leukocyte recruitment [13].

To test whether H_2_O_2_ is required for injury-induced axon growth, we blocked H_2_O_2_ production and monitored axon regeneration after fin amputation by downregulating the primary enzyme generating H_2_O_2_ in the larval fin, Dual oxidase 1 (Duox1), using a previously characterized morpholino (*duox1*-MO) (Figures 5A, S5A) [13]. Injecting this morpholino into embryos prevented the promotion of axon activity by fin amputation (23.89±3.29 µm, *n* = 11, ** *p*<0.01) (Figure 6B,F, Video S7). Interestingly, fin regeneration was also compromised in *duox1* morphants, potentially reflecting a role for axon innervation in fin regeneration, similar to limb regeneration in amphibians [4]. Treating amputated morphant larvae with 1.5 mM H_2_O_2_ for 12 h rescued the deficit in axon reinnervation observed in the morphants (102.3±5.6 µm, *n* = 5, ** *p*<0.01) (Figure 6C,F, Video S8). Due to the toxicity of prolonged H_2_O_2_ treatment, we unfortunately could not assess whether such rescued morphants also regenerated their fins. Blocking H_2_O_2_ production with the *duox1*-MO did not affect growth and retraction induced by axotomy alone (Figure 6D), suggesting that cell-intrinsic mechanisms through which axotomy induces axon activity may be regulated by different pathways. To minimize the possibility that *duox1*-MO toxicity inhibited axon growth following axotomy, we repeated this experiment with co-injection of a morpholino targeting *p53*, which inhibits apoptosis [28], as was done in a previous study with the *duox1*-MO [13]. Like in larvae injected with *duox1*-MO alone, axon growth promotion by amputation was blocked in larvae injected with both *p53*-MO and *duox1*-MO, compared to larvae injected with *p53*-MO alone (Figures 6F, S5B–D), supporting the idea that the *duox1*-MO's effect on regeneration is not due to cellular toxicity. Together these results indicate that Duox1-mediated H_2_O_2_ production is necessary for the promotion of injury-induced axon growth.

**Figure 7:**
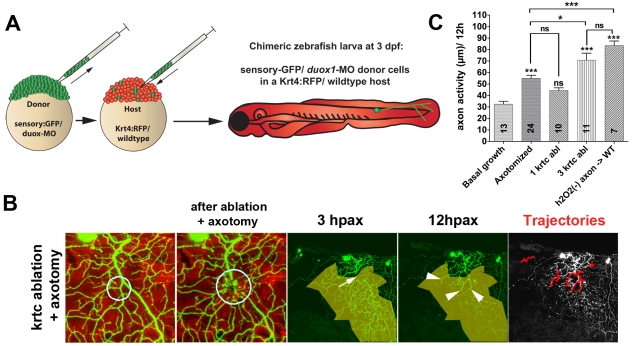
H_2_O_2_ produced by damaged keratinocytes is sufficient to promote axon regeneration. (A) Diagram of the procedure for creating chimeric larvae to test where Duox1 functions to promote axon regeneration: cells from a *duox1*-MO injected sensory:GFP transgenic donor were transplanted into a wildtype Krt4:RFP transgenic host. GFP-labeled Rohon-Beard sensory neurons were thus deficient in Duox1 function, while RFP-labeled keratinocytes were wildtype. (B) Ablation of ≥3 wildtype keratinocytes (circle) and axotomy of a nearby *duox1*-morphant RB axon in the upper trunk region (arrow) promoted regeneration of severed axon branches (arrowheads) and reinnervation of denervated territory (shaded area) (see also Video S9). (C) Quantification of axon activity in chimeric embryos after keratinocyte ablation and axotomy, compared to basal growth (same as in Figure 1E), axotomy alone (same as in Figure 3D), and keratinocyte ablation (same as in Figure 4E). Sample size for each group is indicated by the numbers in the bars. Error bars represent the standard error of the mean. For statistical analyses, we performed one-way ANOVA and either Dunnett's post-test to compare individual groups to controls (asterisks above bars indicate significance compared to control, the first column in graph) or Bonferroni's post-test to compare individual groups with each other (as indicated by brackets, *p* = *ns*>0.05, * *p*<0.05, *** *p*<0.001).

To determine where Duox1 is required to promote axon regeneration, we created genetic chimeras by transplanting cells at the blastula stage from donor embryos injected with *duox1*-MO into uninjected host embryos (Figure 7A). Donor embryos were transgenic for a somatosensory GFP reporter (sensory:GFP) and host embryos were transgenic for a keratinocyte RFP reporter (Krt4:RFP; previously termed Krt8) [27],[29]. At larval stages, we ablated wildtype RFP-labeled keratinocytes in these chimeras and axotomized nearby *duox1* morphant peripheral sensory arbors (*n* = 7 RB neurons, Figure 7B, Video S9). Keratinocyte ablation in these animals significantly promoted axon regeneration, demonstrating that Duox1 is required non-autonomously to achieve the full level of axon growth promotion by keratinocyte ablation (Figure 7C). This result also verified that the ability of the *duox1*-MO to block regeneration was not due to morpholino toxicity in the neuron.

The promotion of axon growth by H_2_O_2_ could in principle result from the direct activation of axon growth or the repression of axon growth inhibitors, such as those that arise after initial growth stages to stabilize axonal structure [24]. To address this issue, we examined regeneration at 30 hpf, when axons are developing and repellants are presumably not present (Figure S6). Axotomy at this stage increased axon activity (** *p*<0.01), but the addition of 3 mM H_2_O_2_ to developing 30 hpf larvae did not further increase activity when compared to untreated larvae. Conversely, knockdown of *duox1* did not significantly change axon activity after fin amputation in 30 hpf larvae (WT versus *duox1*-MO, *p* = *ns*>0.05). These results support the notion that H_2_O_2_ acts by blocking axon growth inhibition, since it only influences regeneration at stages when inhibitors are present. Interestingly, a study in chick showed that axon growth promotion by skin wounds was also only effective at late developmental stages [1].

H_2_O_2_ promotes immune cell recruitment to wounds in developing fruit fly embryos [12] and during early inflammatory responses to fin amputation in larval zebrafish [13]. To test whether inflammation and axon growth are linked or independent effects of H_2_O_2_ signaling, we assessed axon growth in homozygous *cloche* mutants, which lack blood cells [30], and macrophage recruitment in larvae injected with *ngn1*-MO, which lack somatosensory neurons [31]. Amputation promoted axon growth in the absence of blood (91.16±10.44 µm, *n* = 9; Figure 6F, Video S10) and macrophages homed to the wound in the absence of sensory neurons (1.5±0.42 macrophages expressing lysC:GFP at the wound margin within 1 h of amputation, *n* = 6), similar to wildtype (1.0±0.43 macrophages at the wound margin within 1 h of amputation, *n* = 7, *p* = *ns*>0.05; Figure S7), indicating that these two processes are independent of each other.

**Figure 8:**
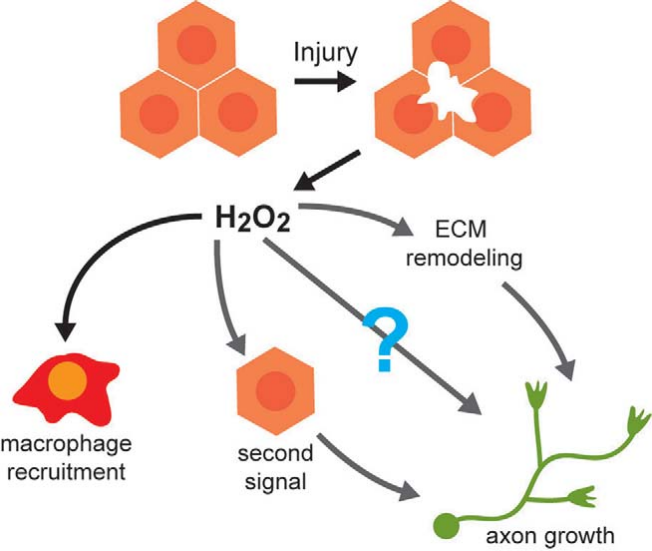
Skin injury and H_2_O_2_ promote peripheral axon regeneration. H_2_O_2_ is generated in response to keratinocyte injury and elicits two independent responses to injury: (1) the recruitment of macrophages to the wound margin [13], and (2) the promotion of peripheral sensory axon growth in the skin. H_2_O_2_'s axon growth-promoting role requires neither leukocytes nor H_2_O_2_ production in neurons themselves. H_2_O_2_ may be promoting axon regeneration by eliciting a second signal (most likely from keratinocytes) that in turn acts on axons, remodeling the extracellular matrix to allow axon growth, or by directly acting on axons, perhaps by blocking their response to inhibitors.

Our results demonstrate that skin injury promotes the growth of axons near the wound, an effect that is mediated by H_2_O_2_ (Figure 8). Keratinocyte ablation and genetic chimera experiments suggested that the axon growth-promoting effects of H_2_O_2_ require its production in keratinocytes. Similarly, in axolotl and chick, wound epidermis attracts axons [1],[2] and damage to human skin co-cultured with rat dorsal root ganglia promotes regeneration of axons at the dermal/epidermal interface [32]. It will be interesting to determine whether H_2_O_2_ also plays a role in these phenomena. Intriguingly, H_2_O_2_ improves hippocampal neurite outgrowth in culture [33]. In *C. elegans*, a mutation in *pxn-2*, which encodes an extracellular peroxidase, improves regeneration of mechanosensory axons [34]. In zebrafish, H_2_O_2_ may be signaling directly to axons, altering the extracellular matrix, or eliciting a second signal from keratinocytes to promote axon growth, but does not require leukocytes (Figure 8). Assessing whether application of H_2_O_2_ to somatosensory neurons in culture can improve axon growth, as has been reported for hippocampal neurons in culture [33], could help resolve whether H_2_O_2_ acts directly or indirectly on axons to influence their regeneration.

In summary, we have found that wounded epidermis promotes somatosensory axon regeneration in zebrafish larvae and that H_2_O_2_ is a critical mediator of this effect. Since this effect does not require the presence of leukocytes, we propose that H_2_O_2_ plays two independent roles during wound healing: promoting axon growth and mediating leukocyte recruitment. Thus, one signaling molecule emitted from injured tissue helps coordinate wound healing with functional recovery of skin.

## Materials and Methods

### Fish Lines and Maintenance

Zebrafish embryos were obtained from Nacre [35], AB (wildtype), Line Mü4435_64 [26], *cloche* (clo^m39^) [30], lysC:GFP [36], sensory:GFP [29], and islet2b:GFP [25] fish. Embryos and larvae were treated with 0.15 mM Phenylthiourea (PTU) to prevent pigment formation.

### Transmission Electron Microscopy (TEM)

TEM was performed according to Rieger & Köster, CSH Protocols Vol. 2 (doi:10.1101/pdb.prot4772, 2007).

### Zebrafish Larval Fin Amputation and Laser Axotomy

#### Fin amputation

Fin amputations were performed according to methods in Kawakami et al. [20]. Briefly, before amputation larvae were anesthetized in 0.01% Tricaine (Sigma, St. Louis, MO) and placed in a petri dish coated with 1.5% agarose. The distal one-third of the fin (posterior to the notochord) was amputated using a sterile syringe needle.

#### Axotomy

Two-photon laser axotomy was performed using a Zeiss LSM 510 META microscope system with a Chameleon laser. Details are described elsewhere [10].

### Time-Lapse Microscopy

Zebrafish larvae were anesthetized in 0.01% Tricaine and mounted in a sealed chamber in 1.2% low-melting agarose (Sigma, St. Louis, MO). Details of the mounting and imaging techniques are described elsewhere [10]. Larvae were imaged for 12 h using a 20× air objective. Stacks were scanned every 30 min in 3 µm intervals. Imaging was performed with 6–10 larvae per session on an LSM 510 confocal microscope (Zeiss) with an automated stage and Multitime software. Larvae were maintained at 28.5°C using a stage heater. Maximum intensity projections of confocal stacks were compiled using Zeiss software and further processed using Adobe Photoshop, NIH open source software Image J 1.34S (Abramoff, NIH Open Source software ImageJ, 2004), and Quick Time Player 7 Pro. For time-lapse imaging of peripheral sensory axon regeneration in H_2_O_2_ solution, 0.005%–0.01% H_2_O_2_ (1.5–3 mM) was added to the larval media 1 h prior to axotomy. Larvae were maintained in H_2_O_2_ solution for 3 or 12 h of time-lapse imaging. See also Figure S1B for timeline of experiments.

### Plasmid Construction

All transgenes were constructed using the Gateway (Invitrogen) tol2kit created by the lab of Chi-Bin Chien [37].

*Tol2CREST3-Gal4VP16-14xUAS-EGFP*: The somatosensory neuron-specific CREST3 enhancer [38] was cloned into the 5′ Gateway vector (p5E), Gal4VP16-14xUAS [39] into the middle vector (pME), and EGFP-SV40pA into the 3′ vector (p3E). Elements were recombined together with the Tol2 destination vector (pDESTTol2). *Tol2CREST3-LexA-LexAop-EGFP*: LexAVP16-SV40pA and four copies of the *LexAop* [40] were cloned into the middle Gateway vector (pME) and recombined with p5E-CREST3 and p3E-EGFP-SV40pA to generate *Tol2CREST3-LexA*-4xLexAop-EGFP.

### Plasmid and Morpholino Injections

Approximately 15 pg of CREST3-Gal4VP16-14xUAS-EGFP or CREST3-LexA-LexAop-EGFP plasmids were co-injected with ∼240 pg of Tol2 [41] transposase mRNA into 1-cell stage embryos of wildtype AB or Nacre strains or into the Gal4-UAS muscle reporter line Tg(Mü4435_64) [26], respectively. A similar amount of CREST3-Gal4VP16-14xUAS-EGFP was co-injected with 10 pg of Krt4:RFP and Tol2 transposase mRNA for keratinocyte ablations. To knock down expression of *p53* [28], *duox1* [13], and *ngn1* [31], 50 nM of each modified antisense oligonucleotide was injected into 1-cell stage embryos.

### Genotyping *duox1* Morpholino-Injected Embryos by RT-PCR

Knockdown of *duox*1 by morpholino injection was verified with RT-PCR, using published primers [13]. Ten larvae at 3 dpf were pooled for RNA isolation and subsequent RT-PCR (see also Figure S5A).

### Determination of Optimal H_2_O_2_ Concentration for Larval Experiments

To determine the sublethal concentration of H_2_O_2_ (Fisher Biotech, 30% in water) to use in larval experiments, we identified the maximum concentration at which 100% of larvae were viable for a minimum of 12 h. Groups of five larvae were incubated in serial dilutions of H_2_O_2_ from 0.003% to 30% and viability was assessed 12 h later. The EC50 was determined to be ∼0.03% (Figure S4). Larvae survived without any morphological abnormalities at 3 mM H_2_O_2_ (0.01%) or less. For rescue experiments, we used a lower concentration of H_2_O_2_ (1.5 mM) to maintain optimal viability.

### H_2_O_2_ Detection

To detect the presence of H_2_O_2_ after amputation or ablation, 5 µM of the H_2_O_2_ sensor pentafluorobenzenesulfonyl fluorescein (Santa Cruz Biotechnology) was added 1 h prior to injury. Larvae were exposed to the sensor throughout the imaging procedure up to 12 h. Fluorescence was detected at 488/505 nm.

### Chimera Analysis

To create chimeras between wildtype and *duox1*-morphants, a few blastula cells (1,000–cell stage) were transplanted from sensory:GFP transgenic embryos injected with *duox1* morpholino into Krt4:RFP wildtype transgenic embryos. Larvae were screened for sensory-specific GFP expression (Duox1-negative neurons) and red fluorescence in keratinocytes (H_2_O_2_-positive skin). Axons were axotomized and imaged as described above.

### Macrophage Quantification

Macrophages were imaged for 12 h in lysC:GFP [36]/islet2b:GFP [25] double-transgenic zebrafish larvae (78 hpf), which were either wildtype or injected with 50nM of *ngn1* morpholino [31] to inhibit sensory neuron development. New macrophages that arrived within 1 h after amputation at the amputation margin were counted and compared between both groups, similar to [13].

### Measurement of Axon Activity, Linear Growth Distance, and Reinnervation of Denervated Tissue

Axon activity was measured by tracing the movements of the 10 axon tips that grew most over a 12 h time window using Image J 1.34S and the Image J Manual Tracking software plugin (F. Cordelires, Institut Curie, Orsay, France). Projected images were adjusted for movement of the specimen, using the Image J StackReg plugin (P. Thévenaz, Swiss Federal Institute of Technology, Lausanne, Switzerland). Measurements were made from projections of 24 time points recorded at 30 min intervals, assuming that axon tips move in a two-dimensional plane. A minimum of 10 axon tips per arbor and specimen were traced. The linear distances of axon growth were quantified using the Zeiss LSM 510 software and ImageJ analysis tool by measuring the distance between the growth cone position in the first (1 h) and last (12 h) stack. To quantify reinnervation of denervated territory, NeuroLucida software (Microbrightfield, Williston, VT) was used to generate tracings of individual Rohon-Beard axons in the fin skin from confocal stacks at 30 min (first recorded time point) and 12 h (last recorded time point). These tracings were overlaid and length measurements were used to quantify the percentage of the axon that entered denervated territory. To minimize distortion caused by developmental growth, images were aligned at the closest shared branch point proximal to the site of axotomy. Statistical analyses were performed using Prism 4 (GraphPad Software Inc.). Unpaired, two-tailed Student's *t*-tests were used for comparisons of two groups (Figures 1E and S2). One-way ANOVA and Dunnett's (comparing groups to a control group) or Bonferroni's (comparing groups to one another) post-tests were performed as indicated in each figure. Significance was set to *p*<0.05. All graphs show the standard error of the mean.

### Axon Density Calculations

Confocal images were loaded into ImageJ software and converted to 8-bit images. A binary image was created and the mean pixel values in a 50×50 µm field in the distal fin portion measured to determine the axon density.

### Quantification of Growth and Retraction

Images were exported as tiff files from the LSM software (Zeiss) and loaded into the ImageJ software. Axon tips were traced as described above and individual movements were designated as growth or retraction within each 30 min interval. The total length of growth and retraction for each arbor was calculated for a 12-h period and a mean value of all traced axon tips derived (*n* = 4 axon tips/4 axons = 16 tracings total).

### Quantifying Degenerating Axon Fragment Size

The detached distal portions of axotomized axons were traced using Neurolucida software (MBF Bioscience) to determine the total combined length of all the branches in the detached arbor. The length was plotted against axon activity of the parent axon during the regeneration phase (12 h).

### Quantification of Escape Behavior

Larvae were placed in a petri dish and tapped with an insect pin at the distal tip of the caudal fin and escapes were recorded. Two groups were compared: wildtype uninjured larvae at 6 dpf and age-matched wildtype larvae whose fins were amputated 3 dpf.

## Supporting Information

Figure S1 Ultrastructure of a larval fin and experimental design. (A) Transmission electron micrograph of a sagittal section through the caudal fin at 48 hpf. The skin consists of two cell layers, the outer periderm (P) and inner epidermal basal cells (B), which are separated by a basement membrane from medially located muscle (M). Magnification is 4,800×. (B) Timeline of experimental procedures. hpf, hours post fertilization.
(15.47 MB TIF)

Figure S2 Quantification of peripheral RB sensory axon reinnervation of denervated territories in the caudal fin. Example tracings are indicated above the bars (see Figure 3 and methods for details). Reinnervation was significantly increased when an axon branch was axotomized after fin amputation as compared to axotomy in non-amputated fins (60.24±13.06 µm versus 14.11±7.02 µm, * *p*<0.05; unpaired, two-tailed Student's *t*-test).
(6.03 MB TIF)

Figure S3 The relative timing of injury and axotomy, but not the size of the severed axon fragment, affects axon regeneration. (A) Quantification of axon regeneration at different time points after axotomy. Axon activity significantly increased when axotomy was performed at 1 hpamp (83.74±3.09 µm, ** *p*<0.01) and 3 hpamp (114.6±7.04 µm, ** *p*<0.01), but axotomy at 6 hpamp (61.20±6.45 µm, *p* = *ns*>0.05) did not significantly promote axon activity when compared to axotomy alone (54.92±2.72 µm) For statistical analyses, we performed one-way ANOVA and Dunnett's post-test to compare individual groups to the control group (first column). (B) Correlation between axotomized arbor size and axon activity. The total length of axotomized arbors is plotted as a function of axon activity, showing that axon activity did not correlate with the size of axotomized arbors. hpamp, hours post amputation; Ax, axotomy.
(0.34 MB TIF)

Figure S4 Survival rates of larvae after treatment with H_2_O_2_ for 12 h. Most of the larvae survived at 0.01% (3 mM) or less.
(0.46 MB TIF)

Figure S5 Knockdown of *duox1* blocks the growth-promoting effects of amputation in p53 morphant larvae. (A) RT-PCR showing knockdown of *duox1* wildtype transcript after morpholino injection as in [13]. Arrows point to the relevant bands. (B–D) Time-lapse sequences from 78–90 hpf. The rightmost panel shows axon tip trajectories (red) over the course of the time-lapse; denervated territories are indicated by shaded areas. (B) Enhanced axon growth in a *p53* control-MO-injected larval fin after amputation (dotted line) and reinnervation of denervated territory (shaded area). (C) Co-injection of *p53*-MO and *duox1*-MO prevented axon growth and reinnervation after amputation. (D) Rescue of axon growth inhibition and reinnervation in *p53*-MO/*duox1*-MO double morphants in the presence of 1.5 mM H_2_O_2_. See quantification in Figure 6F.
(5.71 MB TIF)

Figure S6 Quantification of axon behavior at 30 hpf. None of the groups differed significantly from the control group (untreated uninjured: 99.77±4.96 µm versus untreated 3 mM H_2_O_2_: 111.1±2.03 µm, *p* = *ns*>0.05; untreated axotomy: 135.1±4.53 µm versus 3 mM H_2_O_2_ axotomy: 124.1±2.73 µm, *p* = *ns*>0.05; untreated amputated: 132.7±9.43 µm versus *duox1*-MO amputated: 119.6±7.19 µm, *p* = *ns*>0.05). One-way ANOVA and Bonferroni's post-test were used to compare all groups (*p* = *ns*>0.05, ** *p*<0.01).
(2.93 MB TIF)

Figure S7 Quantification of new macrophages at the wound margin within 1 h after amputation did not reveal a significant difference between wildtype and *neurogenin* 1-morphants, which lack sensory neurons (unpaired, two-tailed Student's *t*-test; *p* = *ns*>0.05).
(0.35 MB TIF)

Video S1 RB axon activity in the caudal fin of an uninjured zebrafish larva. Tracings in the second part of the video show axon tip trajectories.
(2.02 MB MPG)

Video S2 RB axon activity in the non-amputated caudal fin of an axotomized zebrafish larva.
(0.56 MB MOV)

Video S3 RB axon activity in the caudal fin of an amputated zebrafish larva.
(3.43 MB MOV)

Video S4 RB axon activity in the caudal fin of a zebrafish larva after severing an axon branch adjacent to the amputation wound.
(2.03 MB MOV)

Video S5 RB axon activity in the caudal fin of a zebrafish larva after severing an axon branch distant from the amputation wound.
(2.66 MB MOV)

Video S6 RB axon activity after axotomy in a non-amputated caudal fin in the presence of 3 mM H_2_O_2_.
(2.64 MB MOV)

Video S7 RB axon activity in an amputated caudal fin of a *duox*1 morphant.
(2.85 MB MOV)

Video S8 RB axon activity in an amputated fin of a *duox*1 morphant in the presence of 1.5 mM H_2_O_2_.
(0.49 MB MOV)

Video S9 Chimeric zebrafish larva in which a *duox1*-morphant RB axon in the upper trunk was severed following ablation of ≥3 wildtype RFP-labeled keratinocytes.
(0.75 MB MPG)

Video S10 RB axon activity in the caudal fin of a homozygous *cloche* ^−/−^ mutant, which lacks all blood cells.
(2.95 MB MOV)

## Abbreviations

dpf: days post fertilization
*duox1*: dual oxidase 1
GFP: green fluorescent protein
H_2_O_2_: hydrogen peroxide
hpamp: hours post amputation
hpax: hours post axotomy
hpf: hours post fertilization
MO: morpholino
*ngn1*: neurogenin 1
RB: Rohon-Beard
WT: wildtype
