# Supplementary figures and images for: Hydrogen Peroxide Promotes Injury-Induced Peripheral Sensory Axon Regeneration in the Zebrafish Skin
Source: PLoS Biol. 2011 May 24;9(5):e1000621. doi: 10.1371/journal.pbio.1000621 (PMC3101194; doi:10.1371/journal.pbio.1000621)

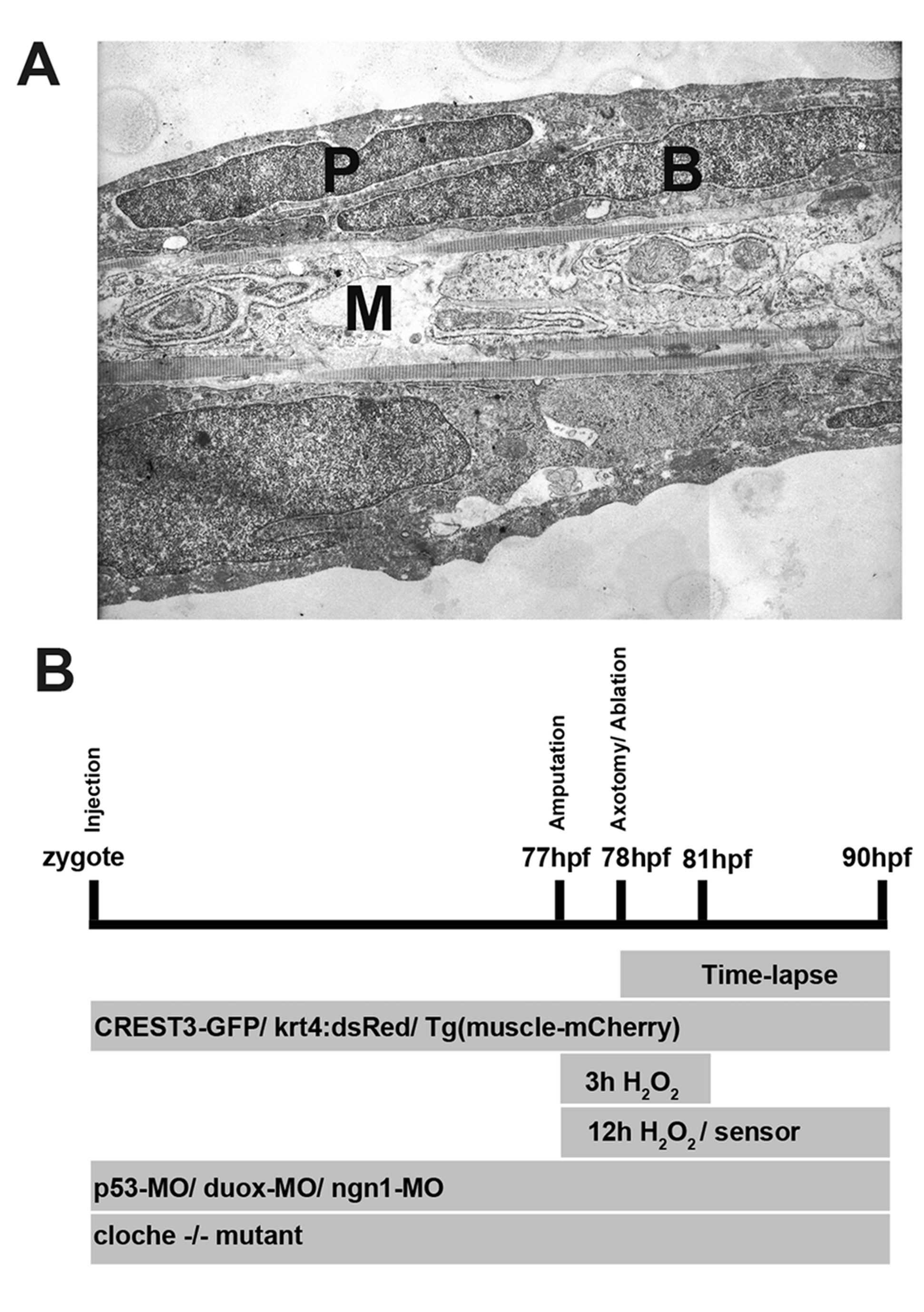

Supplement: Figure S1 — Ultrastructure of a larval fin and experimental design. (A) Transmission electron micrograph of a sagittal section through the caudal fin at 48 hpf. The skin consists of two cell layers, the outer periderm (P) and inner epidermal basal cells (B), which are separated by a basement membrane from medially located muscle (M). Magnification is 4,800×. (B) Timeline of experimental procedures. hpf, hours post fertilization. (15.47 MB TIF) [file pbio.1000621.s001.tif]

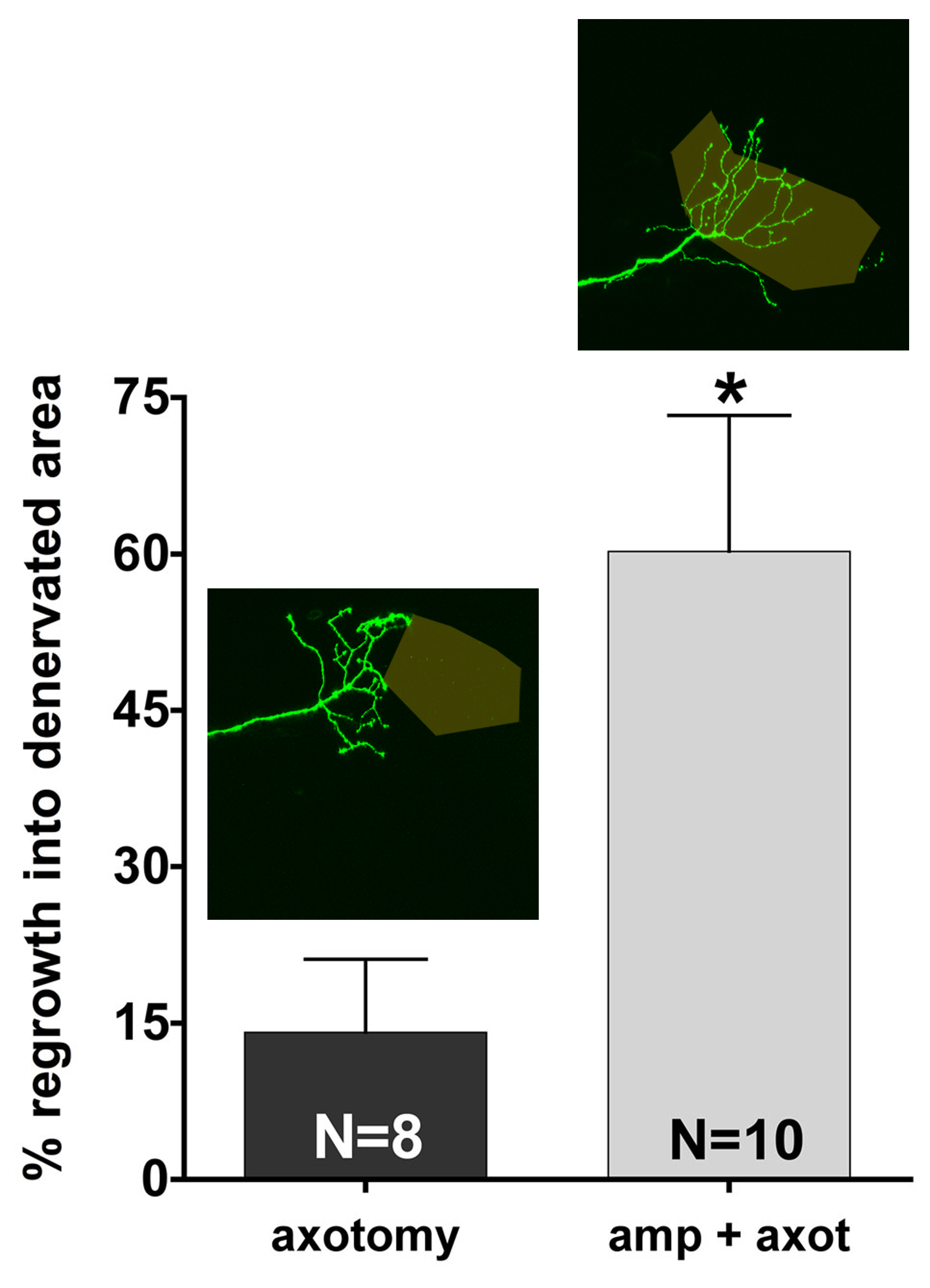

Supplement: Figure S2 — Quantification of peripheral RB sensory axon reinnervation of denervated territories in the caudal fin. Example tracings are indicated above the bars (see Figure 3 and methods for details). Reinnervation was significantly increased when an axon branch was axotomized after fin amputation as compared to axotomy in non-amputated fins (60.24±13.06 µm versus 14.11±7.02 µm, * p<0.05; unpaired, two-tailed Student's t-test). (6.03 MB TIF) [file pbio.1000621.s002.tif]

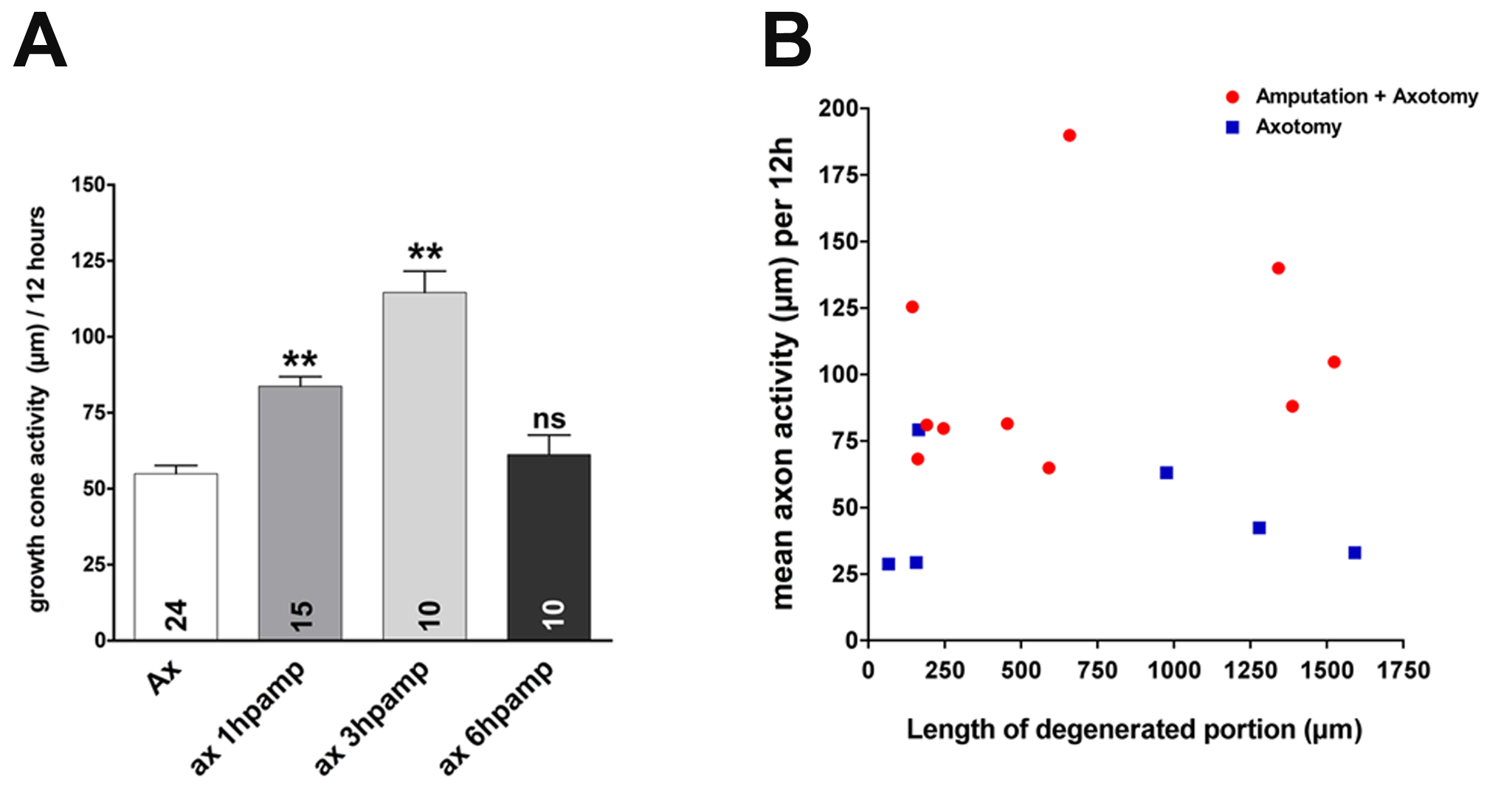

Supplement: Figure S3 — The relative timing of injury and axotomy, but not the size of the severed axon fragment, affects axon regeneration. (A) Quantification of axon regeneration at different time points after axotomy. Axon activity significantly increased when axotomy was performed at 1 hpamp (83.74±3.09 µm, ** p<0.01) and 3 hpamp (114.6±7.04 µm, ** p<0.01), but axotomy at 6 hpamp (61.20±6.45 µm, p = ns>0.05) did not significantly promote axon activity when compared to axotomy alone (54.92±2.72 µm) For statistical analyses, we performed one-way ANOVA and Dunnett's post-test to compare individual groups to the control group (first column). (B) Correlation between axotomized arbor size and axon activity. The total length of axotomized arbors is plotted as a function of axon activity, showing that axon activity did not correlate with the size of axotomized arbors. hpamp, hours post amputation; Ax, axotomy. (0.34 MB TIF) [file pbio.1000621.s003.tif]

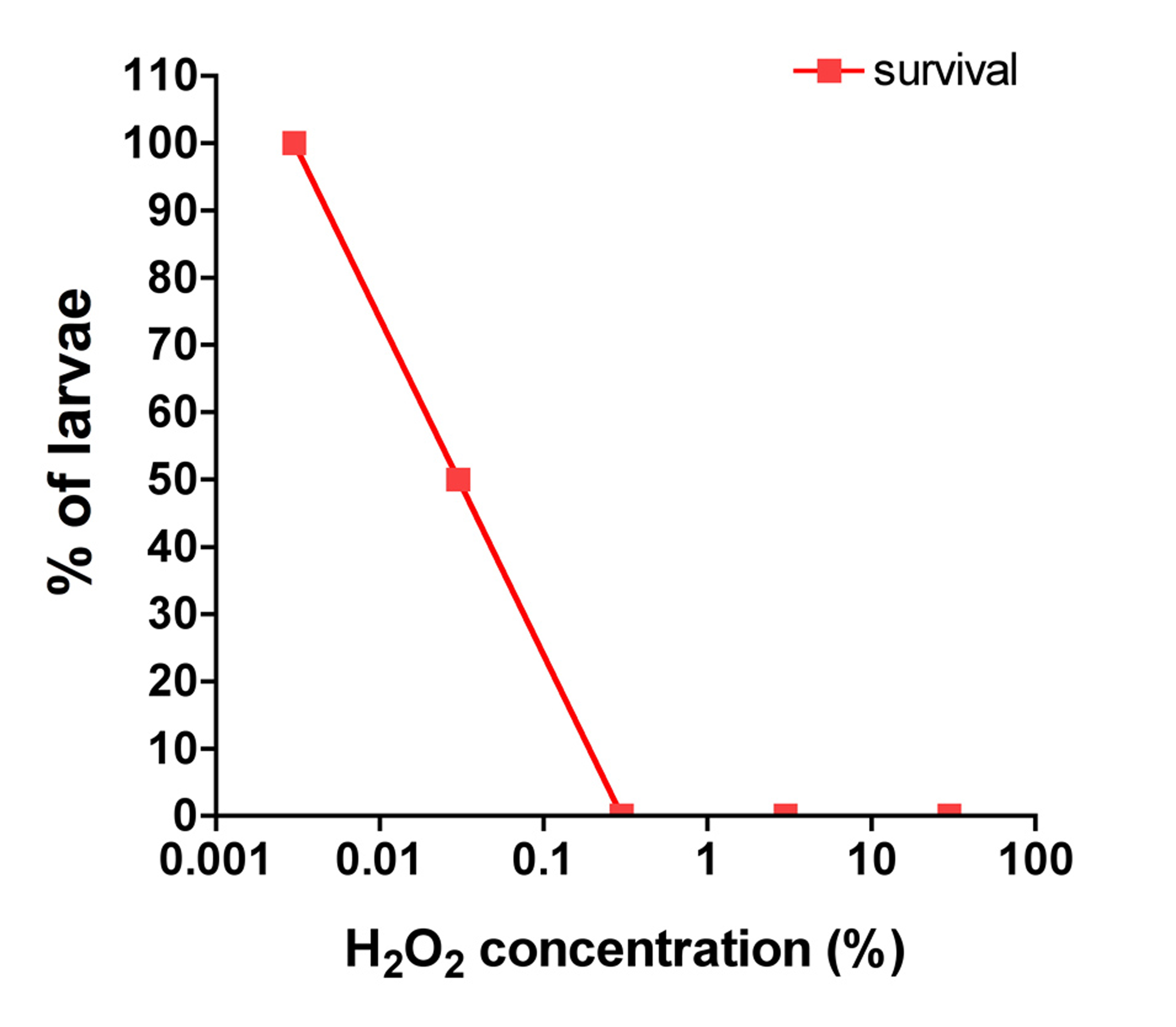

Supplement: Figure S4 — Survival rates of larvae after treatment with H2O2 for 12 h. Most of the larvae survived at 0.01% (3 mM) or less. (0.46 MB TIF) [file pbio.1000621.s004.tif]

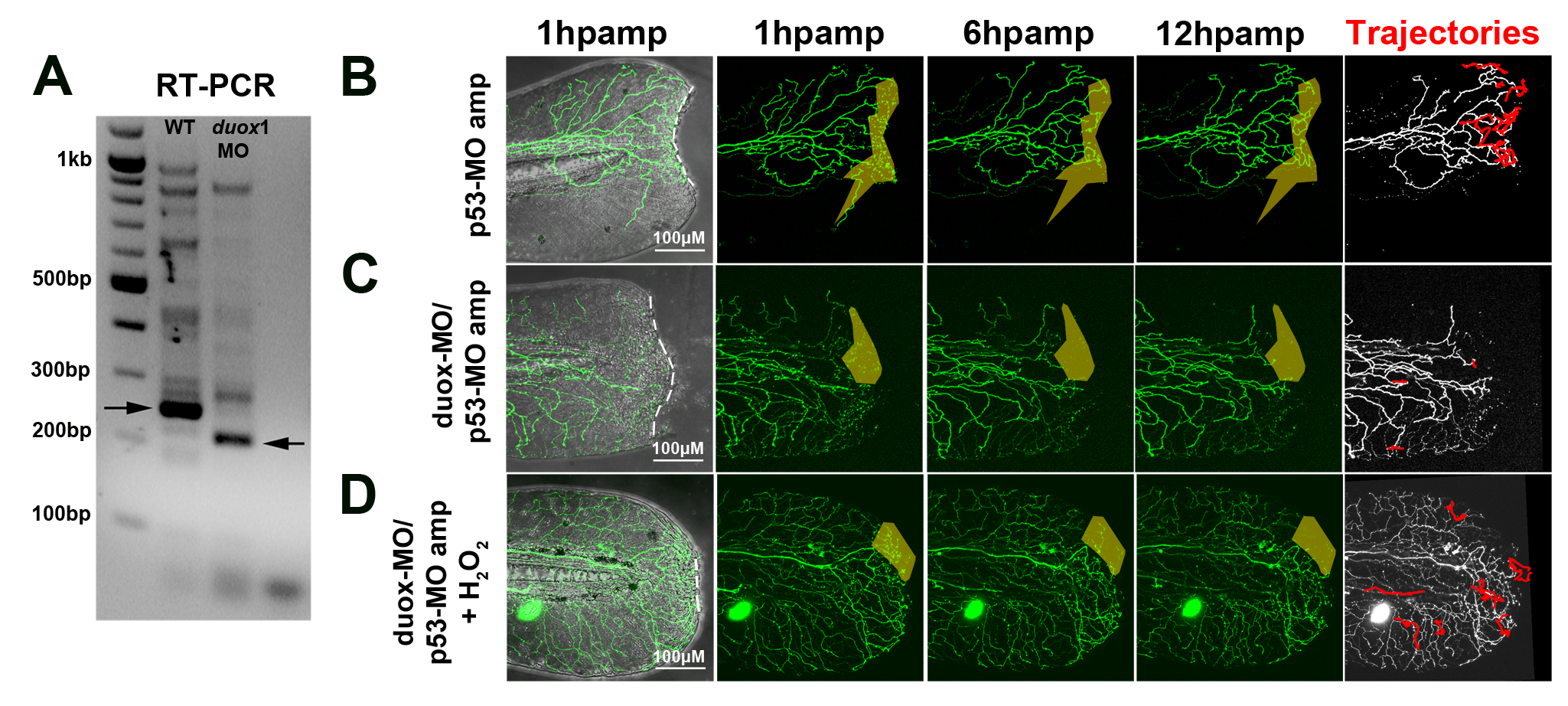

Supplement: Figure S5 — Knockdown of duox1 blocks the growth-promoting effects of amputation in p53 morphant larvae. (A) RT-PCR showing knockdown of duox1 wildtype transcript after morpholino injection as in [13]. Arrows point to the relevant bands. (B–D) Time-lapse sequences from 78–90 hpf. The rightmost panel shows axon tip trajectories (red) over the course of the time-lapse; denervated territories are indicated by shaded areas. (B) Enhanced axon growth in a p53 control-MO-injected larval fin after amputation (dotted line) and reinnervation of denervated territory (shaded area). (C) Co-injection of p53-MO and duox1-MO prevented axon growth and reinnervation after amputation. (D) Rescue of axon growth inhibition and reinnervation in p53-MO/duox1-MO double morphants in the presence of 1.5 mM H2O2. See quantification in Figure 6F. (5.71 MB TIF) [file pbio.1000621.s005.tif]

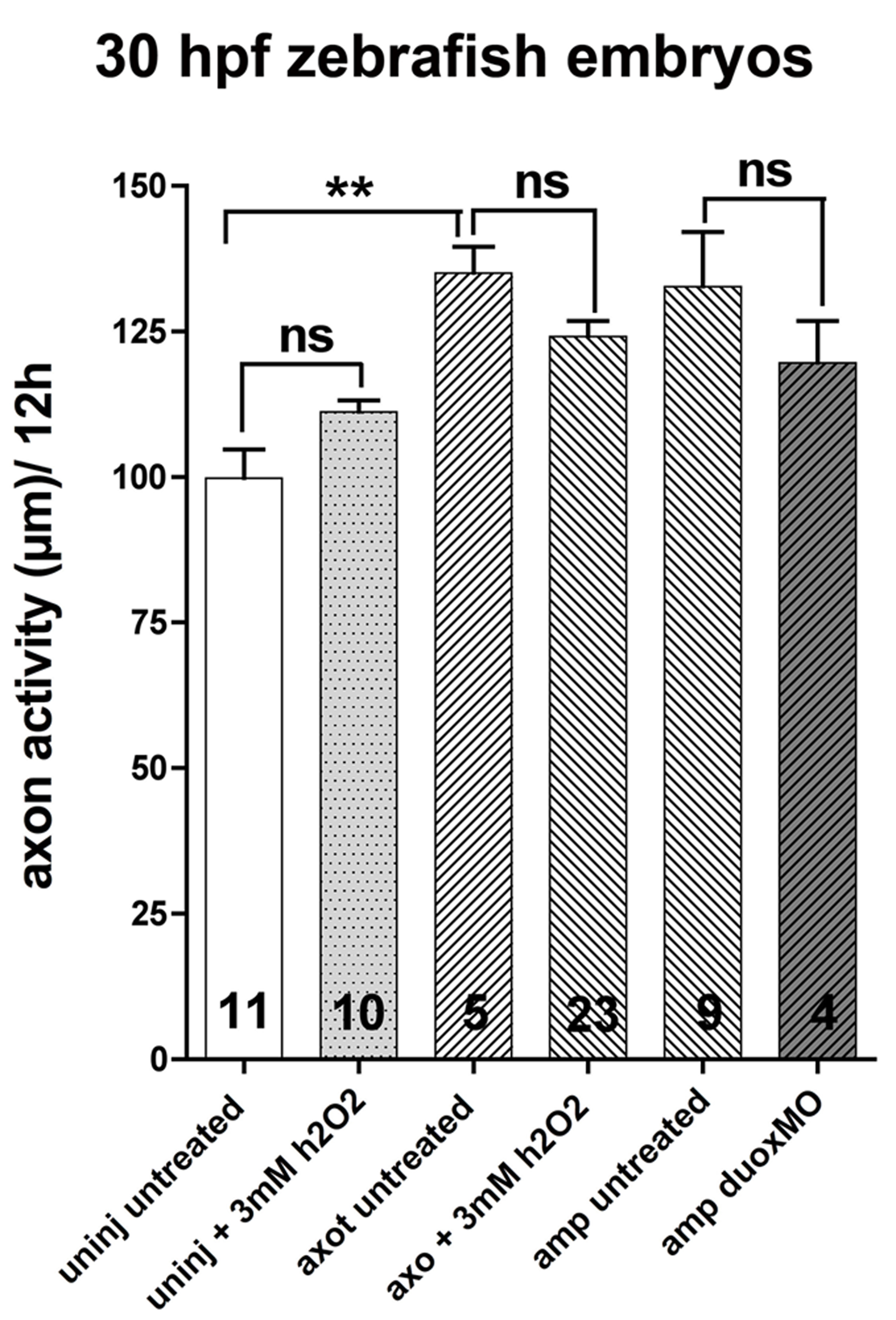

Supplement: Figure S6 — Quantification of axon behavior at 30 hpf. None of the groups differed significantly from the control group (untreated uninjured: 99.77±4.96 µm versus untreated 3 mM H2O2: 111.1±2.03 µm, p = ns>0.05; untreated axotomy: 135.1±4.53 µm versus 3 mM H2O2 axotomy: 124.1±2.73 µm, p = ns>0.05; untreated amputated: 132.7±9.43 µm versus duox1-MO amputated: 119.6±7.19 µm, p = ns>0.05). One-way ANOVA and Bonferroni's post-test were used to compare all groups (p = ns>0.05, ** p<0.01). (2.93 MB TIF) [file pbio.1000621.s006.tif]

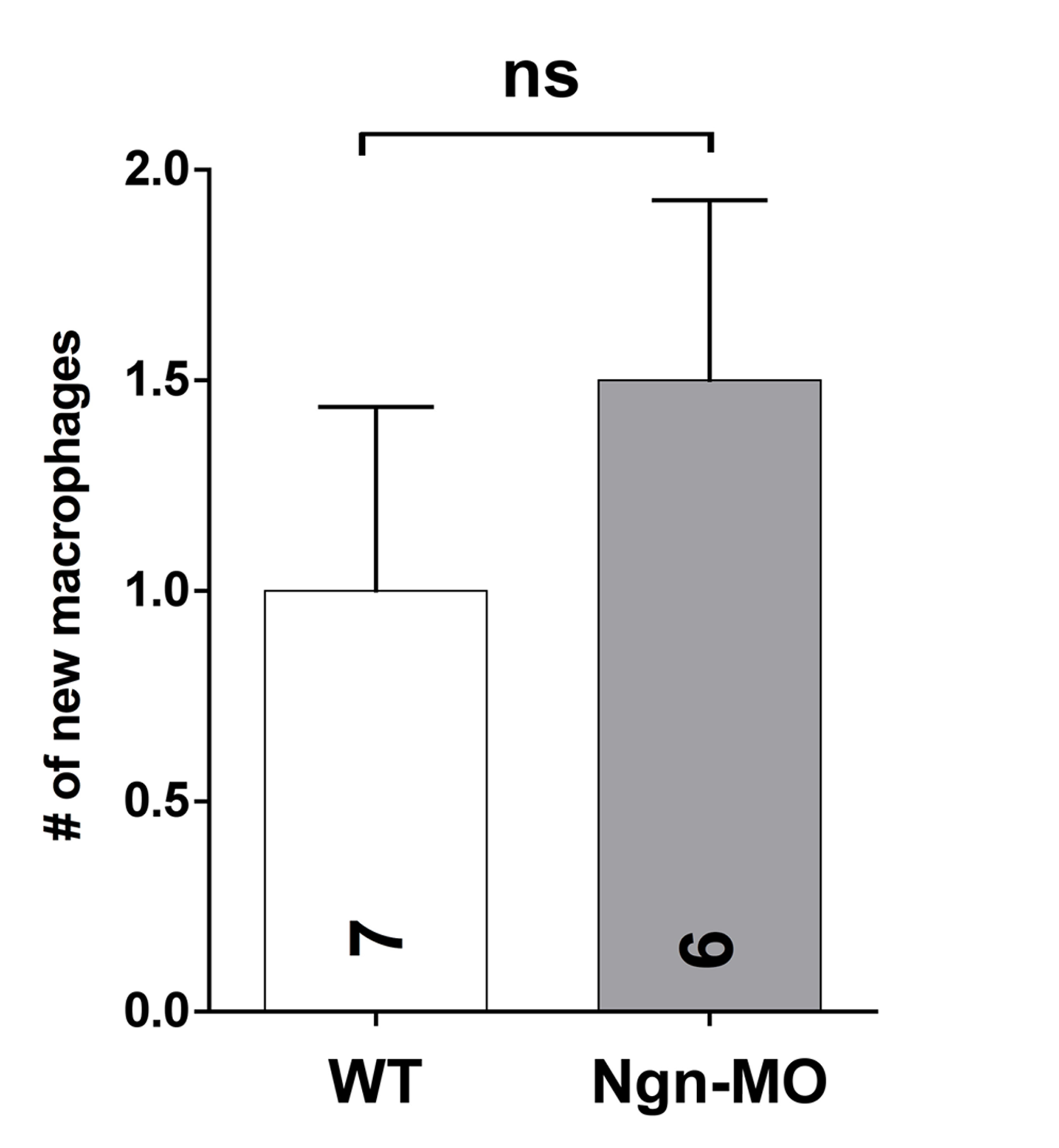

Supplement: Figure S7 — Quantification of new macrophages at the wound margin within 1 h after amputation did not reveal a significant difference between wildtype and neurogenin 1-morphants, which lack sensory neurons (unpaired, two-tailed Student's t-test; p = ns>0.05). (0.35 MB TIF) [file pbio.1000621.s007.tif]
